# Cardiac timing modulates the contribution of compliance to effective arterial elastance

**DOI:** 10.14814/phy2.71112

**Published:** 2026-09-29

**Authors:** Thomas Murchie

**Affiliations:** ^1^ Te Puna Wai Ora, Southern Critical Care Dunedin Hospital Dunedin New Zealand

**Keywords:** arterial compliance, arterial load, effective arterial elastance, ventricular‐arterial coupling, Windkessel

## Abstract

Effective arterial elastance (*E*_a_) is a lumped descriptor of arterial load. The contributions of arterial characteristics to *E*_a_, and their interactions with cardiac timing parameters, have not been fully elaborated. Previous studies have shown that *E*_a_ can be approximated by equations of the form *E*_a_ ≈ *R*/*T* + *k*/*C*, where *R* is either total or peripheral resistance, *T* is cardiac cycle time, and *C* is arterial compliance, but reported values for the coefficient *k* vary substantially, and some analyses suggest that compliance contributes little to *E*_a_. Here, an analytical approximation is derived from Sunagawa's Windkessel expression for *E*_a_ by replacing the exponential term with a truncated Taylor expansion. The resulting approximation predicts that *k* is not constant but varies with cardiac timing, such that *k* ≈ 0.5 (*t*_d_/*T*)^2^, where *t*_d_ is diastolic time. Approximation accuracy was assessed across 693 combinations of resistance, compliance, and heart rate and in a virtual population of 4374 adults derived from a published one‐dimensional vascular model. The linear approximation for *E*_a_ closely tracked the Windkessel expression across the full parameter space (*R* ^2^ > 0.9999, mean absolute percentage error 0.51%). In the virtual population, the approximation showed good agreement with the *E* surrogate *P*_ES_/SV (end‐systolic pressure/stroke volume), surpassing previous approximations. These findings show that the contribution of compliance to *E*_a_ is modulated by cardiac timing and provide a mechanistic explanation for previously reported values of *k*.

## 1 INTRODUCTION

Effective arterial elastance (*E*_a_) is a lumped descriptor of arterial load in the pressure‐volume domain. The respective contributions of arterial resistance and compliance characteristics to *E*_a_, and their interactions with cardiac timing parameters, have not been fully elaborated. Previous analyses of the contribution of compliance to *E*_a_ have produced divergent results.

In a three‐element Windkessel model of the arterial system, *E*_a_ is determined by total systemic resistance (*R*_T_), which is the sum of peripheral resistance (*R*_p_) and characteristic impedance (*Z*_c_), along with arterial compliance (*C*) through the time constant *𝜏* = *R*_p_ *C*, and the durations of systole (*t*_s_) and diastole (*t*_d_), as described by Sunagawa et al. (1983):

(1) $$E_{a}=\frac{R_{T}}{t_{s}+\tau \left(1-e^{-t_{d}/\tau }\right)}$$

A widely recognized surrogate for *E*_a_ is given by the ratio of end‐systolic pressure to stroke volume (*P*_ES_/SV). This ratio and its Windkessel counterpart show good agreement in vivo (Kelly et al., 1992), but both obscure the respective influences of the underlying arterial resistance and compliance characteristics on *E*_a_.

Previous modeling and experimental studies, however, have identified linear approximations of the form:

(2) $$\begin{array}{c} E_{a}=\frac{R}{T}+\frac{k}{C} \end{array}$$

where the contributions of the resistance term *R* (previously represented by *R*_p_ or *R*_T_), and the compliance term *C*, are described more explicitly. While cardiac cycle time (*T*) has a clear physiological meaning, *k* has functioned as a fitted coefficient and empirically derived values for *k* of 0.314 (Segers et al., 2002) and 0.42 (Chemla et al., 2003) have been found. Meanwhile, it has been claimed that *E*_a_ is minimally affected by *C*, that is, that *k* ≈ 0 (Chirinos et al., 2014; Li & Atlas, 2015). The physiological determinants of *k* have not been described analytically.

Here, we show that *k* is not constant but scales with the duration of diastole as a proportion of the cardiac cycle (*t*_d_/*T*). The resulting linear approximation of *E*_a_, following the form of Equation (2), is found to closely track the parent Windkessel equation (Equation 1) across a wide range of inputs and to correlate well with the surrogate *P*_ES_/SV in a 1‐dimensional vascular model. This analysis clarifies how arterial compliance and cardiac timing contribute to *E*_a_, and offers an explanation for the divergent empirical estimates of *k*.

## 2 MATERIALS AND METHODS

A linear approximation for *E*_a_ (Equation 3) was derived analytically from the three‐element Windkessel expression for *E*_a_ reported by Sunagawa (Equation 1) by replacing the exponential term with a polynomial approximation, which gives rise to an approximation for *k* (Equation 4).

The linear approximation for *E*_a_ was assessed against the parent Windkessel equation and against the *E*_a_ surrogate *P*_ES_/SV. These comparisons were used to assess whether the assumptions and truncations introduced during the derivation were reasonable across a relevant range of inputs. Statistical analysis was performed using R statistical software version 4.5.2 (R Project for Statistical Computing).

### 2.1 Comparison with the parent equation

To map the agreement between the linear approximation of *E*_a_ and the parent Windkessel equation, a set of 693 evenly distributed combinations of *R*_T_, *C*, and HR values representing the adult left ventricle and systemic circulation was generated, allowing paired values of *E*_a_ to be compared across this broad parameter space and revealing the sensitivity of error in the approximation to each input variable.

HR values (60, 80 and 100 bpm) were chosen to reflect the adult range in which *E*_a_ might be assessed. The definition of *t*_s_ as ventricular ejection time and *t*_d_ as *T* − *t*_s_ were retained from the Windkessel derivation of *E*_a_ (Sunagawa et al., 1983) and used for both the Windkessel parent equation and the linear approximation in all analyses. While the assumption *𝜏* ≈ *R*_T_ *C* was used to derive the linear approximation, the convention *𝜏* = *R*_p_ *C* was used for the parent equation. Given that ejection time varies with HR, input values for *t*_s_ were adjusted according to the relationship observed during exercise (*t*_s_ (ms) = HR (bpm) × −1.15 + 379) (Mertens et al., 1981).

*R*_T_ values of 0.5–1.75 mmHg s/mL at intervals of 0.125 mmHg s/mL were chosen to encompass the ranges observed in adults in health (Cattermole et al., 2017) and during treatment for septic shock (Monge García et al., 2015) and cardiogenic shock (Mathew et al., 2021). *R*_p_ was modeled as a fixed fraction of total resistance, using *R*_p_ = 0.94 *R*_T_, based on the observation that *Z*_c_ is approximately 5%–7% of peripheral resistance (Westerhof et al., 2009). *C* values of 0.5–2.5 mL/mmHg at intervals of 0.1 mL/mmHg were chosen to encompass the range found by invasive measurement in normotensive and hypertensive adults (Randall et al., 1976). All reference cohorts included male and female subjects.

### 2.2 Comparison with *P*_ES_/SV

To evaluate agreement between the linear approximation of *E*_a_ and the *E*_a_ surrogate *P*_ES_/SV, both quantities were calculated for 4374 simulated adults. This virtual population was generated by Charlton et al., using a one‐dimensional vascular model, described in detail by its authors (Charlton et al., 2019). The model inputs were specified to reproduce age‐appropriate distributions of cardiovascular parameters, simulating a population age range of 25–75 years. Model inputs, arterial pressure and flow data and derived indices are presented in a publicly accessible database (Charlton et al., 2019).

The linear approximation of *E*_a_ was calculated using database‐supplied values for HR, where *T* (s) = 60/HR (bpm), LVET, *R*_T_ (reported in the source dataset as SVR), and *C* (reported there as *C*).

*P*_ES_/SV was calculated using database‐supplied SV, with *P*_ES_ obtained from the aortic root pressure waveform. Since the supplied SV values represent net aortic root flow per cardiac cycle, allowing for a small volume of end‐systolic flow reversal (~0.73 mL), *P*_ES_ was sampled at the end of the reverse flow period, so that *P*_ES_ corresponded to the time at which SV was established. This time point was identified using the publicly available *AorticFlowWave* script used by Charlton et al. to generate the model inflow waveforms, which locates the end of reverse flow at 0.309/0.282 LVET.

Two previous approximations for *E*_a_ were similarly compared with *P*_ES_/SV. These were *E*_a_ ≈ *R*_T_/*T* and Segers et al.'s linear regression model (Segers et al., 2002) *E*_a_ ≈ 1.023 *R*_p_/*T* + 0.314/*C* – 0.127. *R*_p_ was calculated for this analysis as *R*_T_ – *Z*_c_, with characteristic impedance (*Z*_c_) derived from database‐supplied values for aortic root diameter (d), aortic pulse wave velocity (PWV) and blood density (*ρ*) as *Z*_c_ = *ρ* PWV/[π(d/2)^2^].

## 3 RESULTS

The following approximations for *E*_a_ and *k* were derived analytically (see Appendix A):

(3) $$\begin{array}{c} E_{a}=\frac{R_{T}}{T}+0.5\;{\left(\frac{t_{d}}{T}\right)}^{2}\;\frac{1}{C} \end{array}$$

(4) $$\begin{array}{c} k=0.5\;{\left(\frac{t_{d}}{T}\right)}^{2} \end{array}$$

It follows that the influence of *C* on *E*_a_ increases when diastole (*t*_d_) occupies a larger proportion of the cardiac cycle (*T*), which tends to occur when HR is low (Mertens et al., 1981).

### 3.1 Comparison with the parent equation

The linear approximation (Equation 3) closely matched the parent Windkessel equation (Equation 1) across the range of inputs examined (*R* ^2^ > 0.9999, mean absolute percentage error = 0.51%). The truncated Taylor series approximation used here degrades at large values of *t*_d_/*𝜏*, which occurred when HR, *R*_T_ and *C* were simultaneously low. The maximum value of *t*_d_/*𝜏* in this broad parameter space was 2.94. However, the maximum absolute percentage error was 3.96% (Figure 1).

**FIGURE 1:**
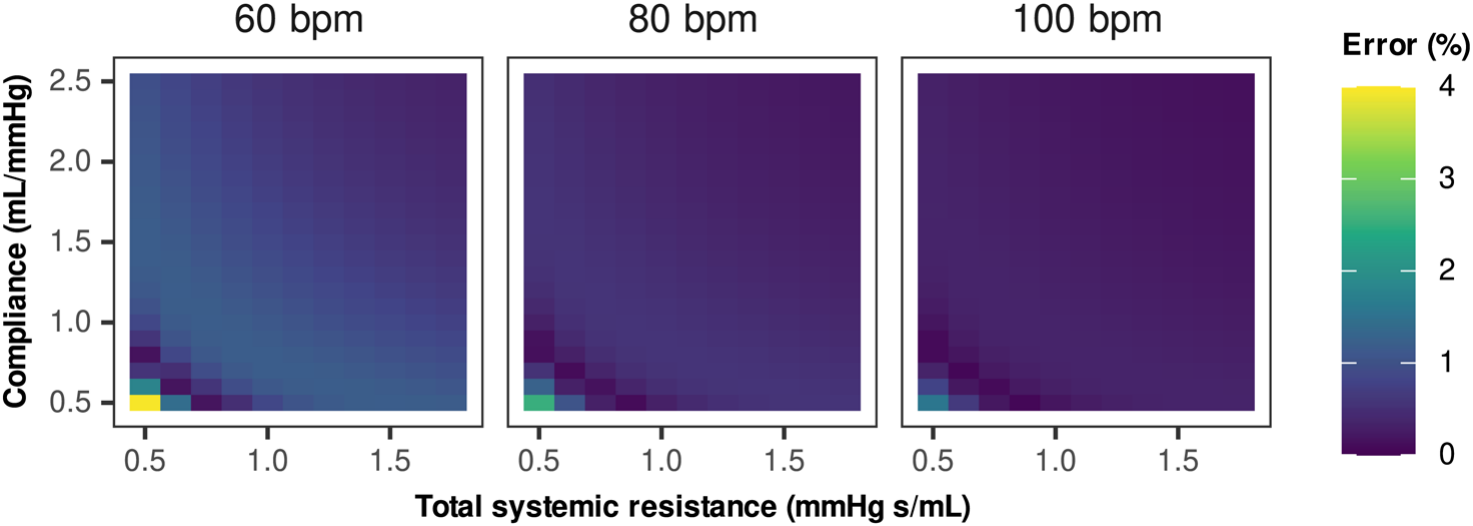
Error of the linear approximation of *E*_a_ relative to the parent Windkessel equation. Absolute percentage error of the linear approximation *E*_a_ ≈ *R*_T_/*T* + 0.5 (*t*_d_/*T*)^2^ 1/*C* relative to the Windkessel equation for *E*_a_ (Equation 1) at 693 points encompassing heart rates of 60, 80 and 100 bpm with 231 combinations of *R*_T_ and *C* at each HR level, and *R*_p_ a fixed fraction of *R*_T_. *C*, arterial compliance; *E*_a_, effective arterial elastance; *R*_p_, peripheral resistance; *R*_T_, total systemic resistance.

### 3.2 Comparison with *P*_ES_/SV

When the linear approximation of *E*_a_ was compared with *P*_ES_/SV across 4374 simulated adults, there was good agreement (Table 1). The maximum value of *t*_d_/*𝜏* among these cases was 1.64, placing them all outside the region in which the Windkessel equation and its linear approximation began to diverge. The previous empirical approximation found by Segers using a constant coefficient *k*, *E*_a_ ≈ 1.023 *R*_p_/*T* + 0.314/*C* ‐ 0.127, and the *k* ≈ 0 approximation *E*_a_ ≈ *R*_T_/*T*, showed progressively poorer agreement with *P*_ES_/SV (Figure 2).

**TABLE 1:** *P*_ES_/SV compared with three approximations of *E*_a_.

	Approximation	*R* ^2^	MAPE	Max APE
A	*k* = 0.5 (*t*_d_/*T*)^2^	0.9980	0.87%	6.81%
B	*k* = 0.314	0.9903	3.80%	14.95%
C	*k* = 0	0.9610	9.99%	25.78%

*Note*: The *E*_a_ surrogate *P*_ES_/SV compared with three variants of the approximation *E*_a_ ≈ *R*/*T* + *k*/*C*: (A) The present approximation, *E*_a_ ≈ *R*_T_/*T* + 0.5 (*t*_d_/*T*)^2^ 1/*C*; (B) the empirical approximation of Segers et al., *E*_a_ ≈ 1.023 *R*_p_/*T* + 0.314/*C* ‐ 0.127, (C) The *k* = 0 approximation *E*_a_ ≈ *R*_T_/*T*. Data were obtained from 4374 simulated adults.

Abbreviations: *E*_a_, effective arterial elastance; MAPE, mean absolute percentage error; Max APE, maximum absolute percentage error.

**FIGURE 2:**
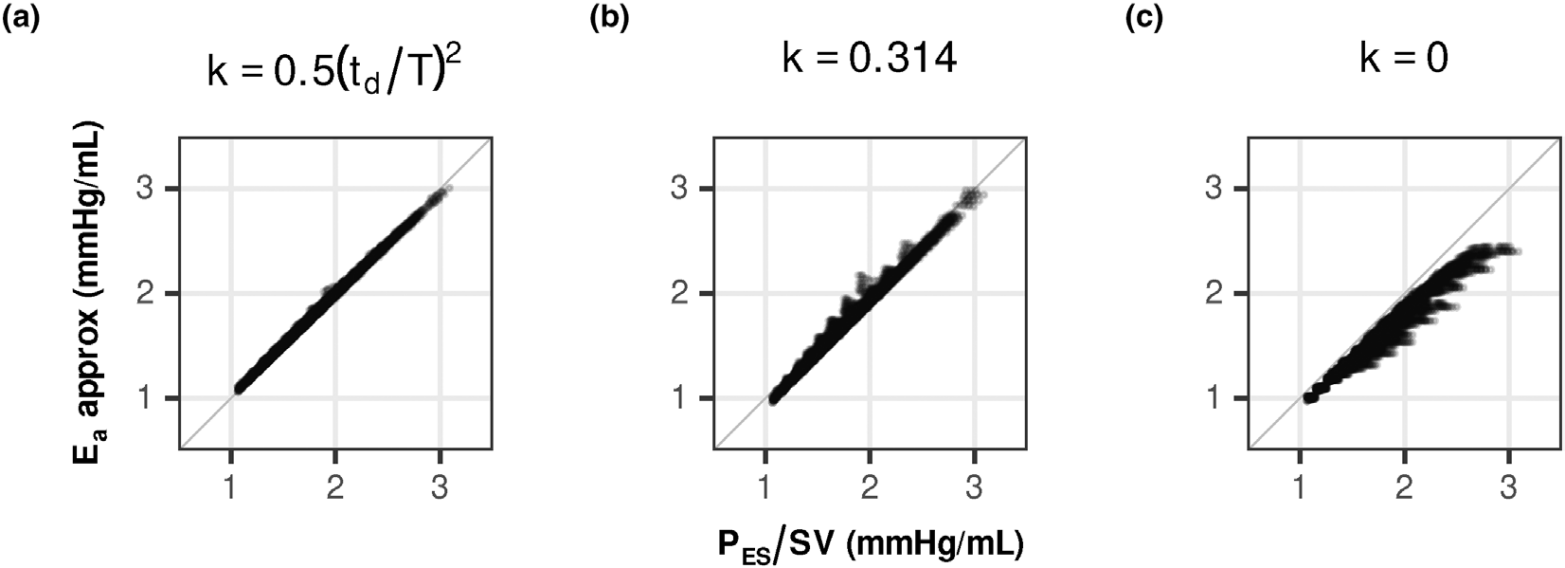
*P*_ES_/SV compared with three approximations of *E*_a_. The *E*_a_ surrogate *P*_ES_/SV compared with three variants of the approximation *E*_a_ ≈ *R*/*T* + *k*/*C*: (a) The present approximation, *E*_a_ ≈ *R*_T_/*T* + 0.5 (*t*_d_/*T*)^2^ 1/*C*; (b) the empirical approximation of Segers et al., *E*_a_ ≈ −0.127 + 1.023 *R*_p_/*T* + 0.314/*C*; (c) The *k* = 0 approximation *E*_a_ ≈ *R*_T_/*T*. Data were obtained from 4374 simulated adults. The gray line represents the line of identity. *E*_a_, effective arterial elastance; *P*_ES_, end‐systolic pressure; SV, stroke volume.

## 4 DISCUSSION

The present analysis shows that the contribution of *C* to *E*_a_ is modulated by cardiac timing and that the coefficient *k* ≈ 0.5 (*t*_d_/*T*)^2^ closely approximates this effect across the range of physiologically relevant parameters examined. Thus, the influence of *C* on *E*_a_ increases as the fraction of the cardiac cycle occupied by diastole increases.

Previous analyses (Chirinos et al., 2014; Li & Atlas, 2015) have suggested that compliance contributes little to effective arterial elastance because the Windkessel expression can be approximated as *E*_a_ ≈ *R*_T_/*T*. This reduction follows from truncating the Taylor series expansion of the exponential expression after the linear term: *𝜏*(1 – e^−*t*d/*𝜏*^) ≈ *𝜏*(*t*_d_/*𝜏*), which cancels *𝜏*, and therefore *C*, from the resulting expression. The present analysis, using a higher‐order approximation, gives a linear expression for *E*_a_ that takes the form previously identified empirically by Segers and Chemla. This approximation, which retains *C* and a leading‐order approximation for its coefficient *k*, showed substantially closer agreement with the parent Windkessel expression and with the *E*_a_ surrogate *P*_ES_/SV across the range of inputs examined. Thus, analytical approximations that reduce *E*_a_ to *R*_T_/*T* and empirical models of the form *E*_a_ ≈ *R*/*T* + *k*/*C* correspond with different orders of approximation.

Conceptually, the factor *k*/*C* can be understood as accounting for the portion of pressure‐volume load not already accounted for by *R*_T_/*T*. Since *E*_a_ ≈ *P*_ES_/SV and *R*_T_/*T* ≈ MAP/SV, *k*/*C* ≈ (*P*_ES_ − MAP)/SV. Therefore, *k* approximates two separate timing‐dependent factors that relate *C* to this excess pressure per unit of SV: the fraction of SV stored in the arterial compartment at end‐systole, rather than lost to systolic peripheral runoff (dV/SV), which approaches zero as *t*_d_/*T* approaches zero; and the fraction of the associated arterial compartment pressure change (dV/*C*) that lies above MAP. The latter depends on the temporal weighting of the arterial pressure decay, which diminishes as *t*_d_/*T* approaches zero, causing MAP and *P*_ES_ to converge regardless of dV.

The effect of cardiac timing on the relationship between *C* and *E*_a_ is a factor that may be significant for empirical work. Since *t*_d_/*T* is known to vary substantially with HR (Mertens et al., 1981), the relationship between *C* and *E*_a_, observed across a population containing natural variation in HR, may not reflect the contribution of *C* to *E*_a_ for each individual unless the timing‐dependence of this contribution is taken into account. If *t*_d_/*T* covaries systematically with *C*, the compliance‐dependent term *k*/*C* might vary minimally across a population, despite the physical dependence of *E*_a_ on *C*.

This analysis examines the interpretation of vascular Windkessel parameters in the range of the systemic circulation, but does not address uncertainty in their estimation, does not include parameters in the range of the pulmonary circulation, and does not examine the relationships between derived arterial load expressions and pressure‐flow relationships in vivo.

## AUTHOR CONTRIBUTIONS

**Thomas Murchie:** Conceptualization; data curation; formal analysis; investigation; methodology; project administration; resources; software; visualization.

## CONFLICT OF INTEREST STATEMENT

Thomas Murchie: No perceived or potential conflict of interest.

## ETHICS STATEMENT

Ethics approval was not required, since this study analysed de‐identified published data and did not collect any new human or animal data.

## Data Availability

Source data for this study are openly available at https://doi.org/10.5281/zenodo.22188467.

## APPENDIX A

A linear approximation for effective arterial elastance (*E*_a_) can be derived by starting with the expression for *E*_a_ described by Sunagawa (A1), where *R*_T_ is the sum of peripheral resistance (*R*_p_) and characteristic impedance (*Z*_c_), *C* is arterial compliance, *T* is the cardiac period, which is the sum of the systolic period (*t*_s_) and diastolic period (*t*_d_), and *𝜏* is the arterial time constant, given by *R*_p_ *C*. Since *Z*_c_ ≪ *R*_p_, *R*_p_ ≈ *R*_T_, allowing the simplifying assumption *𝜏* ≈ *R*_T_ *C*.

(A1)
$$\begin{array}{c} E_{a}=\frac{R_{T}}{t_{s}+\tau \left(1-e^{-t_{d}/\tau }\right)} \end{array}$$

Assuming *t*_d_/*𝜏* is small, the exponential function e^−*t*d/*𝜏*^ is approximated by the first three terms of the Taylor expansion:

$$e^{-t_{d}/\tau }\approx 1-\frac{t_{d}}{\tau }+\frac{t_{d}^{2}}{2\tau^{2}}$$

Rearranging and multiplying by *𝜏*:

$$\tau \;\left(1-e^{-t_{d}/\tau }\right)\approx t_{d}-\frac{t_{d}^{2}}{2\tau }$$

Under the assumption that *𝜏* ≈ *R*_T_ *C*:

$$\tau \;\left(1-e^{-t_{d}⁄\tau }\approx t_{d}-\frac{t_{d}^{2}}{2R_{T}C}\right)$$

Substituting this approximation into the denominator of (A1) and simplifying:

$$E_{a}\approx \frac{R_{T}}{T-t_{d}^{2}⁄2R_{T}C}$$

Using a first‐order approximation for 1/(*T* − *t*_d_^2^/(2*R*_T_ *C*)) under the assumption that *t*_d_^2^/(2*R*_T_ *C* ) ≪ *T*:

$$E_{a}\approx R_{T}\;\left(\frac{1}{T}+\frac{t_{d}^{2}}{2R_{T}CT^{2}}\right)$$

Rearranging gives:

(A2)
$$\begin{array}{c} E_{a}\approx \frac{R_{T}}{T}+0.5\;{\left(\frac{t_{d}}{T}\right)}^{2}\frac{1}{C} \end{array}$$

It follows that:

(A3)
$$\begin{array}{c} k\approx 0.5\;{\left(\frac{t_{d}}{T}\right)}^{2} \end{array}$$

## References

[phy271112-bib-0001] Cattermole, G. N. , Leung, P. Y. , Ho, G. Y. , Leung, P. Y. M. , Ho, G. Y. L. , Lau, P. W. S. , Chan, C. P. Y. , Chan, S. S. W. , Smith, B. E. , Graham, C. A. , & Rainer, T. H. (2017). The normal ranges of cardiovascular parameters measured using the ultrasonic cardiac output monitor. Physiological Reports, 5(6), e13195. 10.14814/phy2.13195 PMC5371563 28320891

[phy271112-bib-0002] Charlton, P. H. , Mariscal Harana, J. , Vennin, S. , Li, Y. , Chowienczyk, P. , & Alastruey, J. (2019). Modeling arterial pulse waves in healthy aging: A database for in silico evaluation of hemodynamics and pulse wave indexes. American Journal of Physiology. Heart and Circulatory Physiology, 317(5), H1062–H1085. 10.1152/ajpheart.00218.2019 PMC6879924 31442381

[phy271112-bib-0003] Chemla, D. , Antony, I. , Lecarpentier, Y. , & Nitenberg, A. (2003). Contribution of systemic vascular resistance and total arterial compliance to effective arterial elastance in humans. American Journal of Physiology. Heart and Circulatory Physiology, 285(2), H614–H620. 10.1152/ajpheart.00823.2002 12689857

[phy271112-bib-0004] Chirinos, J. A. , Rietzschel, E. R. , Shiva‐Kumar, P. , de Buyzere, M. L. , Zamani, P. , Claessens, T. , Geraci, S. , Konda, P. , de Bacquer, D. , Akers, S. R. , Gillebert, T. C. , & Segers, P. (2014). Effective arterial elastance is insensitive to pulsatile arterial load. Hypertension, 64(5), 1022–1031. 10.1161/HYPERTENSIONAHA.114.03696 25069668

[phy271112-bib-0005] Kelly, R. P. , Ting, C. T. , Yang, T. M. , Liu, C. P. , Maughan, W. L. , Chang, M. S. , & Kass, D. A. (1992). Effective arterial elastance as index of arterial vascular load in humans. Circulation, 86(2), 513–521. 10.1161/01.cir.86.2.513 1638719

[phy271112-bib-0006] Li, J. K. , & Atlas, G. (2015). Left ventricle‐arterial system interaction in heart failure. Clinical Medicine Insights: Cardiology, 9(Suppl 1), 93–99. 10.4137/CMC.S18742 PMC4479180 26124691

[phy271112-bib-0007] Mathew, R. , di Santo, P. , Jung, R. G. , Marbach, J. A. , Hutson, J. , Simard, T. , Ramirez, F. D. , Harnett, D. T. , Merdad, A. , Almufleh, A. , Weng, W. , Abdel‐Razek, O. , Fernando, S. M. , Kyeremanteng, K. , Bernick, J. , Wells, G. A. , Chan, V. , Froeschl, M. , Labinaz, M. , … Hibbert, B. (2021). Milrinone as compared with dobutamine in the treatment of cardiogenic shock. New England Journal of Medicine, 385(6), 516–525. 10.1056/NEJMoa2026845 34347952

[phy271112-bib-0008] Mertens, H. M. , Mannebach, H. , Trieb, G. , & Gleichmann, U. (1981). Influence of heart rate on systolic time intervals: Effects of atrial pacing versus dynamic exercise. Clinical Cardiology, 4(1), 22–27. 10.1002/clc.4960040106 7226587

[phy271112-bib-0009] Monge García, M. I. , Guijo González, P. , Gracia Romero, M. , Gil Cano, A. , Oscier, C. , Rhodes, A. , Grounds, R. M. , & Cecconi, M. (2015). Effects of fluid administration on arterial load in septic shock patients. Intensive Care Medicine, 41(7), 1247–1255. 10.1007/s00134-015-3898-7 26077088

[phy271112-bib-0010] Randall, O. S. , Esler, M. D. , Calfee, R. V. , Bulloch, G. F. , Maisel, A. S. , & Culp, B. (1976). Arterial compliance in hypertension. Australian and New Zealand Journal of Medicine, 6(suppl 2), 49–59. 10.1111/j.1445-5994.1976.tb03323.x 1067827

[phy271112-bib-0011] Segers, P. , Stergiopulos, N. , & Westerhof, N. (2002). Relation of effective arterial elastance to arterial system properties. American Journal of Physiology. Heart and Circulatory Physiology, 282(3), H1041–H1046. 10.1152/ajpheart.00764.2001 11834502

[phy271112-bib-0012] Sunagawa, K. , Maughan, W. L. , Burkhoff, D. , & Sagawa, K. (1983). Left ventricular interaction with arterial load studied in isolated canine ventricle. American Journal of Physiology, 245(5 Pt 1), H773–H780. 10.1152/ajpheart.1983.245.5.H773 6638199

[phy271112-bib-0013] Westerhof, N. , Lankhaar, J. W. , & Westerhof, B. E. (2009). The arterial Windkessel. Medical & Biological Engineering & Computing, 47(2), 131–141. 10.1007/s11517-008-0359-2 18543011

